# Developing Activated Carbon Veil Electrode for Sensing Salivary Uric Acid

**DOI:** 10.3390/bios11080287

**Published:** 2021-08-20

**Authors:** Maria A. Bukharinova, Natalia Yu. Stozhko, Elizaveta A. Novakovskaya, Ekaterina I. Khamzina, Aleksey V. Tarasov, Sergey V. Sokolkov

**Affiliations:** Scientific and Innovation Center of Sensor Technologies, Department of Physics and Chemistry, Ural State University of Economics, 8 Marta St., 62, 620144 Yekaterinburg, Russia; m.a.buharinova@usue.ru (M.A.B.); lizanovakovskaya@list.ru (E.A.N.); xei260296@mail.ru (E.I.K.); tarasov_a.v@bk.ru (A.V.T.); ssokolkov@yandex.ru (S.V.S.)

**Keywords:** electrochemical nonenzymatic sensor, carbon veil, carbon paper, 3D material, electrochemical activation, uric acid, saliva, non-invasive analysis, antioxidant activity

## Abstract

The paper describes the development of a carbon veil-based electrode (CVE) for determining uric acid (UA) in saliva. The electrode was manufactured by lamination technology, electrochemically activated and used as a highly sensitive voltammetric sensor (CVE_act_). Potentiostatic polarization of the electrode at 2.0 V in H_2_SO_4_ solution resulted in a higher number of oxygen and nitrogen-containing groups on the electrode surface; lower charge transfer resistance; a 1.5 times increase in the effective surface area and a decrease in the UA oxidation potential by over 0.4 V, compared with the non-activated CVE, which was confirmed by energy dispersive X-ray spectroscopy, electrochemical impedance spectroscopy, chronoamperometry and linear sweep voltammetry. The developed sensor is characterized by a low detection limit of 0.05 µM and a wide linear range (0.09–700 µM). The results suggest that the sensor has perspective applications for quick determination of UA in artificial and human saliva. RSD does not exceed 3.9%, and recovery is 96–105%. UA makes a significant contribution to the antioxidant activity (AOA) of saliva (≈60%). In addition to its high analytical characteristics, the important advantages of the proposed CVE_act_ are the simple, scalable, and cost-effective manufacturing technology and the absence of additional complex and time-consuming modification operations.

## 1. Introduction

Uric acid (UA) is produced in the human body as a result of the enzymatic decomposition of purine bases and nucleic acids. The level of UA in blood serum, urine and saliva is a biomarker of some systemic diseases [1]. High levels of UA are associated with such disorders as kidney diseases, gout, hypertension, Lesch-Nyhan syndrome, diabetes, cancer, and HIV, and can also accompany obesity [2]. On the contrary, low levels of UA can signal Alzheimer’s disease, multiple sclerosis, and mild cognitive impairment [1]. In this regard, methods of the quick determination of UA in human physiological fluids are attracting widespread interest.

Saliva is a very promising diagnostic matrix. As a biological material for analysis, saliva is more advantageous than plasma or blood serum. Its non-invasive sampling is characterized by cost-effectiveness, reduced complexity of the analysis and lower risk of patient’s infection, which is an obvious benefit. The UA level in saliva of patients with oral diseases is considered to be a biomarker of antioxidant defence against oxidative stress [3]. Monitoring salivary levels of UA is important both for diagnostic purposes and for treatment programs. Some researchers report a linear correlation between the level of UA in blood serum and saliva [1,4,5].

A variety of methods can be used to measure UA in biological fluids, including fluorescent [6], chromatographic with mass spectrometry, electrochemical and fluorescence detection [1,7,8], enzymatic colorimetric [9], capillary electrophoresis with electrochemical detection [10,11], and electrochemical [12,13]. These methods have both benefits and limitations. Chromatographic methods requires complex and expensive equipment and the pre-processing of samples; they are time-intensive and cannot be applied in point-of-care diagnostics. As for enzymatic colorimetric methods, due to enzyme instability, its activity may decrease over time, which is the reason for lower sensitivity of the analysis. In addition, for the reaction of UA and uricase, optimal pH and temperature must be maintained [14]. In order to obtain reliable results, the method of capillary electrophoresis often requires the modification of the inner surface of the capillary, temperature control, and washing before each new measurement, which increases the complexity of the analysis. The shortcomings of this method also include low sensitivity during UA determination and insufficient reproducibility.

Electrochemical methods, characterized by simplicity, high sensitivity, and selectivity, serve as powerful analytical tools for monitoring UA levels. Among the advantages of electrochemical methods is miniaturization and the possibility for its use in on-site measurements. To date, numerous electrochemical sensors and biosensors have been developed to determine UA in biological (clinical) samples [15,16,17,18]. Different enzymes (uricase, horseradish peroxidase and their combinations), polymers (redox, conducting, ion-exchange, molecularly imprinted), and nanomaterials (nanoparticles, nanotubes, nanosheets, nanoflakes) are used for sensor fabrication, which reduces electrochemical overvoltage to increase the rate of electron transfer and improve sensitivity and selectivity of measurements. At the same time, since electrochemical signals depend on the source of the enzyme and its degradation over time [15], the thickness of the polymer film [16,17], the size and structure of nanomaterials [18], the conditions for modifying agent fabrication, must be strictly controlled, which is not always possible in reality. Most sensors have a modifying composite layer that may contain various materials. Sometimes the process of creating a multicomponent modifier and composite layers is multi-staged and time-consuming [19,20,21,22] and requires the use of harmful solvents [21,23]. In this regard, the search for original approaches and new electrically conductive materials with highly developed active surfaces, which can be used for creating simple and highly sensitive sensors for UA determination is relevant.

Carbon veil (CV), or carbon paper (CP), is considered as an advanced material for creating new electrochemical sensors. Since this material has a well-developed three-dimensional (3D) surface, we think that the term “carbon veil” is preferable, as the term “carbon paper” is more associated with a two-dimensional (2D) material. CV is a thin non-woven fabric, with multiple fibers randomly intertwined and bonded by a light binder, which ensures a porous structure of the material with high surface area. CV has various properties: good electrical conductivity, electromagnetic interference shielding, static dissipation, chemical and thermoresistance, and low specific gravity. These properties have made CV a popular material in fuel cells, aerospace, and civil construction [23]. Due to biological inertia and radioparency CV can be exploited for applications in medicine (e.g., prostheses, implants, X-ray equipment) [24].

CV is an advanced material for manufacturing highly sensitive (bio)sensors with the use of amperometric or voltammetric methods of signal recording [25]. Currently, voltammetric and amperometric sensors based on CV have been developed for the determination of nitrites [26,27], ascorbic acid [28], and ketoprofen [29]. Due to the CV effective surface, when developing highly sensitive electrochemical sensors, it has become possible to stop using additional carbon-based modifiers, such as carbon nanotubes, graphene, graphene oxide [26,28]. Tarasov et al. [30] used CV in combination with the single-sided hot lamination technology to manufacture a potentiometric sensor system for evaluating the antioxidant activity of human skin. The advantages of the hot lamination technology are simplicity, speed, and scalability; it allows to control the shape and size of manufactured CV-based electrodes and their integration in miniaturized and portable devices.

The present paper focuses on the development of a simple, highly sensitive voltammetric carbon veil-based sensor, fabricated by using the hot lamination technology, for determining salivary UA.

## 2. Materials and Methods

### 2.1. Chemicals and Reagents

Chemicals Na_2_HPO_4_·12H_2_O (JSC Vecton, St. Petersburg, Russia), KH_2_PO_4_ (NevaReaktiv Ltd., St. Petersburg, Russia), NaHCO_3_ (JSC ChemReactivSnab, Ufa, Russia), NaOH (JSC ChemReactivSnab, Ufa, Russia), KCl (Akros Ltd., St. Petersburg, Russia), NaCl (OJSC Mikhailovsky Chemical Reagents Plant, Barnaul, Russia), K_3_[Fe(CN)_6_] (JSC Vekton, St. Petersburg, Russia), K_4_[Fe(CN)_6_]·3H_2_O (AO Reachim Ltd., Moscow, Russia), Cementit universal (Merz+Benteli AG, Niederwangen, Switzerland), acetone (JSC EKOS-1, Moscow, Russia), uric acid (Acros Organics, Geel, Belgium), ascorbic acid (Sigma-Aldrich Co, St. Louis, MO, USA), creatinine (Merc, Darmstadt, Germany), urea (Fluka, Buchs, Switzerland), glucose (JSC Vecton, St. Petersburg, Russia). All reagents were chemically pure and used without additional purification. Working solutions were prepared using deionized water (electrical resistivity—18 MΩcm).

Electrodes were fabricated on the basis of polyacrylonitrile carbon veil (surface density—30 g/m^2^; surface electrical resistivity—8–10 Ω, manufactured by M-Carbo Ltd. (Minsk, Belarus) and polyethylene terephthalate sheets sized 303 × 216 × 0.125 mm (Fellowes Inc., Itasca, IL, USA).

### 2.2. Apparatus

Carbon veil electrodes (CVE) were fabricated utilizing an LM-260iD laminator (Rayson Electrical MFG., Ltd., Foshan, China). Scanning electron microscopy (SEM) images were taken by a Quattro S electron microscope (Thermo Fisher Scientific Inc., Waltham, MA, USA). The analysis of the elemental composition of the CV surface was conducted by energy dispersive X-ray spectroscopy (EDS), utilizing a Quattro S scanning electron microscope and an EDAX Octane Elite detector (AMETEK, Ametek Inc., Berwyn, IL, USA) at an accelerating voltage of 2 kV and a sample current of 1 nA. Electrochemical impedance spectroscopy (EIS) was performed using a μAutolab Type III potentiostat/galvanostat (Metrohm, Herisau, Switzerland). A semi-automatic computerized voltammetric analyzer IVA-5 (IVA, Yekaterinburg, Russia) with a PE-6100 magnetic stirrer and a three-electrode cell was used for cyclic, linear sweep voltammetric and chronoamperometric studies. A CVE was referred as the working electrode; a glassy carbon rod as the auxiliary electrode, and a silver–silver chloride electrode (Ag/AgCl/KCl, 3.5 M) EVL-1M3.1 (JSC Gomel Plant of Measuring Devices, Gomel, Belarus) as the reference electrode. Antioxidant activity (AOA) of saliva samples was measured utilizing a multifunctional potentiometric analyser MPA-1 (IVA, Yekaterinburg, Russia). The planar platinum electrode (IVA, Yekaterinburg, Russia) served as the working electrode and Ag/AgCl/KCl, 3.5 M served as the reference electrode in potentiometric measurements.

### 2.3. Procedures

#### 2.3.1. Electrode Fabrication and Sensor Preparation

The working electrodes were fabricated by applying the hot lamination technology [26,28]. The production process is described below. First, a piece of carbon veil was attached on a polyethylene terephthalate substrate and run through a hot-roll laminator at temperature of 140 °C. Then the resulting substrate with the carbon veil adhesive was cut into over 500 strips, 3 mm wide each. The middle section of the electrode separating the working and contact zones was isolated with a mixture of cementite and acetone (v:v, 1:5). Thus, the area of the working zone of the electrode was 15 mm^2^ (5 × 3 mm). Then the electrode was washed in the water–acetone mixture (v:v, 1:1) by applying constant stirring for 15 min. The obtained electrode was labeled a carbon veil-based electrode (CVE). To activate the CVE electrochemically, it was placed in a solution of 0.05 M of sulfuric acid and kept at different potentials (1.0, 1.6, 2.0 V) for 5 min. Thus, CVE_act_ was prepared.

#### 2.3.2. Sampling and Sample Preparation

Artificial saliva and human saliva were used for sampling. Artificial saliva was prepared following [31] and contained 0.0125 g of NaCl; 0.0964 g of KCl; 0.0654 g of KH_2_PO_4_; 0.02 g of urea and 0.0631 g of NaHCO_3_ in 100 mL of deionized water. Prior to the analysis, salivary pH was changed to 7.0, applying NaOH.

Volunteers, adults aged 25–28 years, provided saliva samples. The samples were collected in the morning following the recommendations [1,32]. Eligible participants (hereinafter referred to as volunteers) were non-smokers, since smoking results in lower levels of UA and AOA in saliva [1]. Volunteers produced unstimulated saliva for 2 min, then it was collected by passive drool into vials. The procedure was repeated three times. Saliva samples were assayed without any additional processing.

#### 2.3.3. Electrochemical Measurements

Phosphate buffer (PB) pH 5–8 was used as background electrolyte for recording UA signal with the linear sweep (LS) voltammetry. LS voltammograms were registered in the potential range from 0.1 V to 0.7 V at a potential scan rate of 0.025–0.300 Vs^−1^ and cyclic voltammograms, from 0.5 V to +1.1 V at 0.05 Vs^−1^. Chronoamperometric measurements were taken at the potential +0.5 V in 0.1 M KCl as background electrolyte. EIS measurements were performed at an ambient temperature, by applying the sine potential of 5 mV and polarization potential of 0.25 V. The frequency range was from 0.04 Hz to 1.0 kHz. EIS spectra were fitted to a Randles-type equivalent circuit.

### 2.4. Statistical Analysis and Data Treatment

The presented results are the average value of multiple (no less than three) measurements. The results were calculated for a confidence level of 0.95. Limits of detection (LOD) and quantification (LOQ) were calculated as 3 SD/b and 10 SD/b, respectively, where SD is the standard deviation of the analytical signal and *b* is the slope of the dependences I_p_ vs C_UA_. The recovery of UA was determined in accordance with IUPAC guidelines [33]. 

## 3. Results

At the beginning of the experiment, in order to determine UA, an attempt was made to use the earlier proposed sensors based on a carbon veil modified by phytosynthesized gold nanoparticles (gr-Au/CVE) [28] and a non-ionogenic surfactant Triton X-100 (TrX100/CVE) [26]. Figure S1 shows the LS voltammograms of 0.1 mM UA on gr-Au/CVE and TrX100/CVE. In comparison with TrX100/CVE, a stronger UA signal at a lower overvoltage was recorded on gr-Au/CVE. In order to achieve a greater effect of a UA signal and lower overvoltage of UA electrooxidation, a new method of treating the carbon veil surface was used, namely, electrochemical activation at high positive potentials. The treatment of carbon materials at high positive potentials enforces the sorption and electrochemical activity of these materials [34,35], which results from the formation of active oxygen-containing groups on the electrode surface. These groups are good mediators of electron transfer.

As can be seen from Figure S1, the peak current of the UA oxidation recorded on the CVE activated at 2.0 V in the sulfuric acid solution was 4 and 2.3 times greater than on TrX100/CVE and gr-Au/CVE, respectively. At the same time, the peak potential on the activated CVE was shifted to the cathodic area by 440 and 100 mV relative to TrX100/CVE and gr-Au/CVE, respectively. Because of the preliminary obtained results, further experiments were focused on the study of the activated CVE and the relevant electrochemical behavior of UA.

### 3.1. CVE Activation

**Figure 1:**
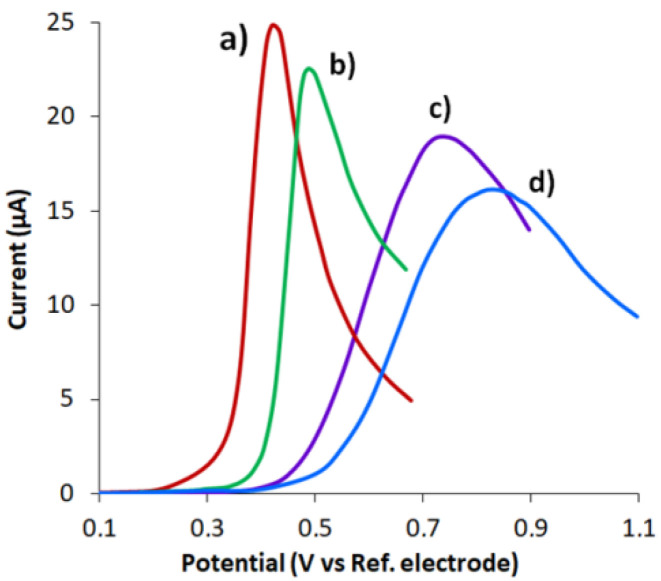
LS voltammograms of 0.1 mM UA for the CVE activated at 2.0 V (**a**), at 1.6 V (**b**) and at 1.0 V (**c**) and non-activated CVE (**d**) in PB pH 6.0. Potential scan rate 0.05 Vs^−^^1^.

Electrochemical activation of CVE was performed at different positive potentials applied for 300 s in a solution of 0.05 M H_2_SO_4_. The choice of the activation potential was judged by the peak current oxidation for 0.1 mM UA in PB and by its potential. According to Figure 1, the highest peak current of UA oxidation and its lowest potential were recorded when the electrode activated at E = 2.0 V was applied (Figure 1a). The potential of this peak was shifted by 0.41 V to the cathodic region relative to the non-activated CVE (Figure 1d), which indicates that the process of UA electrooxidation at CVE_act_ was being accelerated, and the peak current value was 30% more than at the CVE, which may be due to CVE_act_ larger active area (see Section 3.2.).

### 3.2. Characterization of CVE and CVE_act_

CVEs were characterized using SEM, EDS, and electrochemical methods. The morphology of the electrode surface was studied by SEM (Figure S2). As illustrated by Figure S2, the CVE has a three-dimensional porous structure due to thin carbon fibers woven and bonded together by a polymer binder. The diameter of the carbon fibers does not exceed 15 μm.

The elemental analysis of CVEs was carried out with EDS. Figure S3 presents the EDS spectra for non-activated (Figure S3a) and activated CVE at different potentials (Figure S3b,c). As can be seen from the spectra, C, O and N are present on different parts (“fiber” and “binder”) of the CVE and CVE_act_ surfaces. Table 1 gives the results of EDS microanalysis for non-activated and activated CVE at different potentials. It is apparent from Table 1 that an increase in the applied potential of potentiostatic polarization of the electrodes caused a decrease in the weight per volume of C by 21.7% for the “fiber” section on CVE_act_ at 2.0 V relative to CVE, while the weight per volume of O and N went up 55 and 1.8 times, respectively. The activation of poorly conductive “binder” sections did not affect the chemical characterization of the surface. Thus, the electrochemical treatment of the CVE leads to a higher number of surface oxygen and nitrogen-containing groups on electrically conductive fibers. Zhu et al. [34] found that for the most part, the groups such as C=O, C−OH, C−O−C, as well as nitrogen oxides (NO_x_) were present on the electrochemically activated carbon surface. The formation of oxygen- and nitrogen-containing groups leads to the additional bonding of UA with the electrode surface [36].

**Figure 2:**
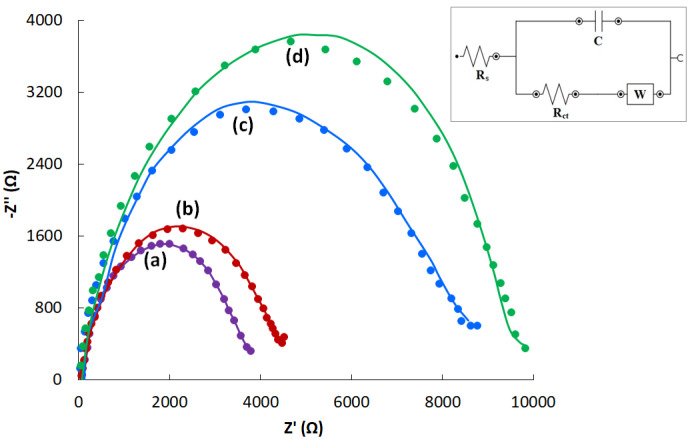
Experimental (points) and fitted (lines) Nyquist plots for CVE_act_ at 2.0 V (**a**), 1.6 V (**b**), 1.0 V (**c**) and CVE (**d**) in 0.1 M KCl containing 1.0 mM [Fe(CN)_6_]^3−/4−^. Insert: the diagram of an Randles equivalent cell.

The charge transport properties of the electrodes were studied using EIS in the presence of 1 mM hexacyanoferrate (II)/(III). The corresponding experimental and fitted Nyquist plots are provided in Figure 2. The insert in Figure 2 presents a diagram of the equivalent electrochemical cell used in the experiment. It includes the electrolyte resistance (R_s_), the capacity (C) of the charge transfer resistance (R_ct_), and the Warburg element (W).

As shown in Figure 2, the diameter of the semicircle of activated CVEs (Figure 2a–c) is smaller than that of the non-activated CVE (Figure 2d). Moreover, the smallest diameter is observed for the CVE activated at 2.0 V (Figure 2a). The obtained results confirm a lower charge transfer resistance, i.e., a higher electron transfer rate for activated CVEs. Good agreement of the experimental and fitted curves in Figure 2 indicates the correct choice of the equivalent cell. The obtained EIS characteristics are presented in Table 2.

The CVE activation led to a lower R_ct_. For the CVE activated at 1.0, 1.6, 2.0 V, R_ct_ went down by 1.3, 2.4, and 2.8 times, respectively, compared to the non-activated CVE, which indicated a better electron transfer. The CVE activated at 2.0 V was used in further studies.

**Figure 3:**
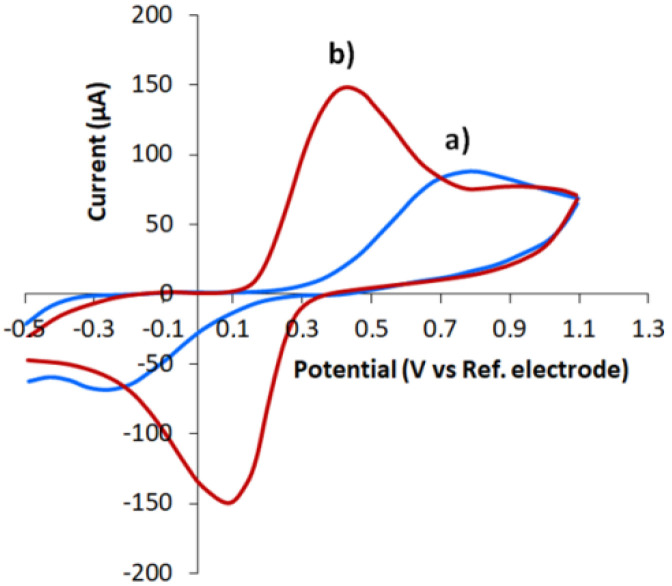
Cyclic voltammograms of 1.0 mM [Fe(CN)_6_]^4−^ on CVE (**a**) and CVE_act_ at 2.0 V (**b**) in 0.1 M KCl. Potential scan rate 0.05 Vs^−1^.

The electrochemical behaviour of the [Fe(CN)_6_]^4−/3−^ Red/Ox pair on CVE and CVE_act_ was studied. As can be seen in Figure 3, there was an increase in [Fe(CN)_6_]^4−/3−^ oxidation/reduction currents and a decrease in the potential difference between the anodic and cathodic peaks for CVE_act_ as compared with the non-activated CVE. In addition, in comparison with the non-activated CVE, a better shape of the peaks for CVE_act_ was observed. Cyclic voltammograms showed that the ratio of the cathodic and anodic currents was 1.01 for CVE_act_ and 0.77 for CVE. The potential difference between the cathode and anode peaks was 0.34 V for CVE_act_ and 1.05 V for CVE.

**Figure 4:**
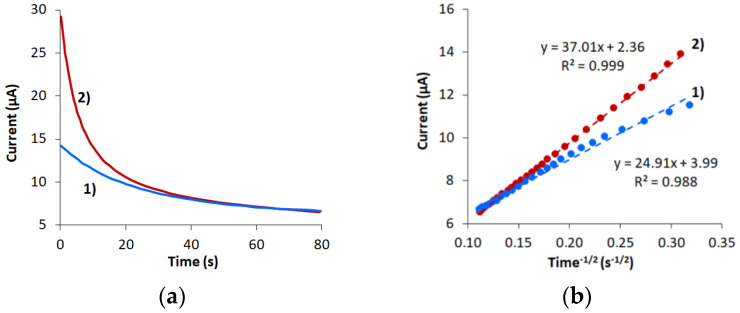
Chronoamperograms obtained at 0.5 V potential on CVE (**1**) and CVE_act_ at 2.0 V (**2**) in 0.1 M KCl containing 1.0 mM K_4_[Fe(CN)_6_] (**a**). Dependencies i = f(t^−^^1/2^) are obtained from chronoamperograms on the corresponding electrodes (**b**).

The obtained results demonstrate that electrochemical reaction of [Fe(CN)_6_]^4−/3−^ Red/Ox pair is irreversible on CVE and reversible on CVE_act_. In this regard, chronoamperometry and the Cottrell equation that is applicable for both reversible and irreversible electrochemical processes were used to measure the effective surface area of the electrodes. For that purpose the chronoamperograms of K_4_[Fe(CN)_6_] oxidation were registered at a potential of 0.5 V at CVE and CVE_act_ (Figure 4a), and the dependence i = f (t^−1/2^) (Figure 4b). As evident from Figure 4b, the slope of the linear dependence i = f(t^−1/2^) for CVE_act_ is approximately 1.5 times higher than for CVE.

The effective surface area of the electrode was computed following the Cottrell equation (Equation (1)):

(1) $$i\;={\;\mathrm{nFAC}}_{0}\frac{\sqrt{D}}{\sqrt{\pi t}}.$$

where n = 1 (the number of electrons); F = 96500 Qmol^−1^; A (cm^2^)—effective surface area; C_0_ (mol cm^3^)—K_4_[Fe(CN)_6_] concentration; D = 7.6 × 10^−6^ cm^2^ s^−1^ (diffusion coefficient); t (s)—time.

The effective area of the non-activated CVE was 16.6 mm^2^, and the area of CVE_act_ was 24.6 mm^2^. Due to electrochemical activation, the degree of coating of the carbon veil with various oxygen-containing functional groups increases, which contributes to a bigger active surface area [35,37].

### 3.3. Effect of pH

**Figure 5:**
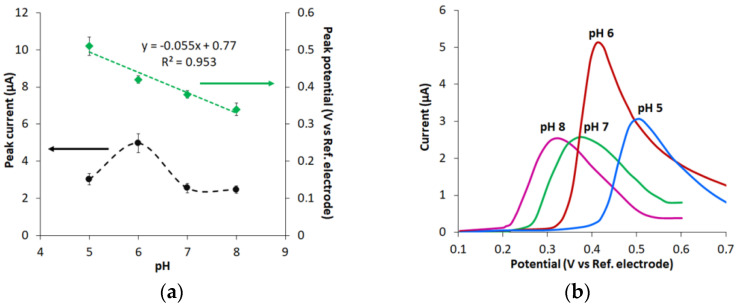
The effect of background electrolyte pH on UA oxidation peak current (I_p_) and peak potential (E_p_) on CVE_act_ at 2.0 V (**a**); and the corresponding LS voltammograms for 0.01 mM UA in PB (**b**). Potential scan rate 0.05 Vs^−^^1^.

The pH value of PB plays a significant role in electrochemical sensing of UA. The effect of background electrolyte pH on electrooxidation of UA at the CVE was studied within the range from 5 to 8 (Figure 5).

The UA oxidation potential decreases linearly with a higher pH, which signals that protons are involved in the electrochemical reaction. The slope of Equation (2) happened to be 0.055 V per pH unit, which suggests that the number of protons and electrons involved in the process of electrooxidation is equal.

E_p_ (V) = (0.770 ± 0.08) − (0.055 ± 0.007) pH, R^2^ = 0.953 (2)

**Scheme 1:**
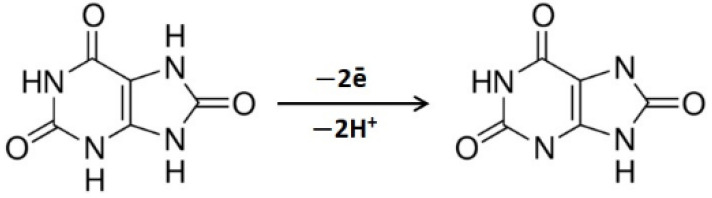
UA electrooxidation.

According to [20,21,22,38], two electrons and two protons participate in UA electrooxidation (Scheme 1).

As can be seen in Figure 5a, the peak oxidation current depends on the solution pH, and it is the highest at pH 6.0. Considering that the UA dissociation constant (pKa) is 5.4–5.75 [36,39], UA is present mostly in the protonated form in pH 6.0 solution and in the acid form in pH > 6.0 solution. The protonated form of UA facilitates the two-electron transfer reaction (Schema 1); moreover the peak current of UA oxidation is the highest. The acid form generates the reaction with one electron transfer [39,40], which causes lower peak oxidation current (Figure 5). If pH < pKa (in our case pH 5.0), UA solubility decreases [41], which leads to lower peak current of UA oxidation. Thus, pH 6.0 was used for further studies. The selected pH 6.0 value is also beneficial for a better separation of the peak potentials of UA and ascorbic acid (interferent) [36].

### 3.4. Effect of Potential Scan Rate

**Figure 6:**
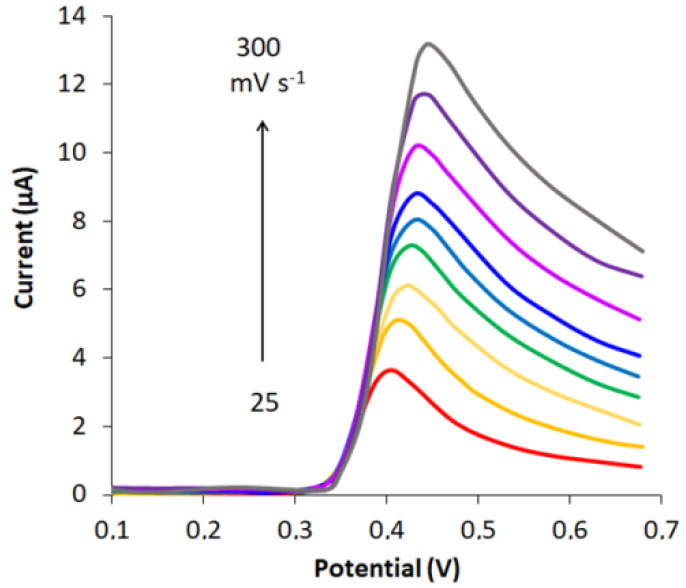
LS voltammograms of 0.01 mM UA on CVE_act_ at 2.0 V in PB pH 6 for different scan rates (25–300 mVs^−1^).

The effect of potential scan rate on peak current and peak potential of UA oxidation was measured in the range from 25 to 300 mVs^−1^ (Figure 6).

As can be seen from Figure 6, a higher scanning rate causes a higher current peak of UA oxidation, and its potential shifts to the anodic region, which indicates that the process of UA electrooxidation on CVE_act_ is irreversible and can be described by Equation (3) (Figure S4a).

E_p_ (V) = (0.015 ± 0.001)lnν + (0.459 ± 0.013), R^2^ = 0.997 (3)

The process of UA electrooxidation on CVE_act_ at 2.0 V is diffusion-controlled, which proves the linearity of the dependence I_p_ = f(ν^1/2^) (Equation (4)). In addition, the slope of the dependence lnI_p_ = f(lnν) (Equation (5)), equal to 0.51, is close to the theoretical value of 0.5 [42], which also confirms the diffusion nature of the process.

I_p_ (μA) = (24.06 ± 0.51) ν^1/2^ (s^1/2^) − (0.35 ± 0.02), R^2^ = 0.996 (4)

lnI_p_ = (0.51 ± 0.02) lnν + (3.16 ± 0.11), R^2^ = 0.997 (5)

### 3.5. Analytical Characteristics of CVE_act_

The peak current of UA increases linearly with an increase in its concentration in the range from 0.09 to 700 μM (Figure S5) and is described by Equation (6):

I_p_ (μA) = (0.36 ± 0.02) C (μM) + (0.076 ± 0.002), R^2^ = 0.995 (6)

The detection limit of the developed sensor was 0.05 μM, the limit of quantitative determination was 0.15 μM. The relative standard deviation of the analytical signal of 0.1 μM UA on one electrode was 4.2 % (n = 5), and for twenty different electrodes 7.4 %.

It was found that a 1000-fold excess of creatinine, urea, glucose, as well as a 10-fold excess of ascorbic acid does not affect the signal of 1 μM of UA (Table S1). According to the literature, the concentration of UA in saliva is considerably higher than the concentration of ascorbic acid. Thus, the level of salivary UA of a relatively healthy person varies from 30 to 180 μM [43], while the level of ascorbic acid is 4 μM [44]. Therefore, the impact of ascorbic acid on the UA determination in saliva can be neglected.

Table 3 presents different sensors that can be used for determining salivary UA. As indicated in Table 3, in comparison with some existing sensors, the proposed sensor has a lower detection limit and a wider linear concentration range. In addition to high analytical characteristics, the important advantages of the developed sensor are a simple and scalable manufacturing technology, the absence of additional complex and time-consuming modification operations. The low cost and good reproducibility of an analytical signal from electrode to electrode make the sensor disposable. Consequently, after-analysis regeneration of the electrode becomes unnecessary.

### 3.6. Salivary UA Determination

The proposed sensor was tested with artificial and human salivary samples. The algorithm of the sample analysis was as follows. First, a background voltammogram was recorded in 9.9 mL of PB pH 6.0. Then 0.1 mL of saliva was added into the electrochemical cell, well mixed, and a linear voltammogram was registered in the potential range from 0.1 to 0.7 V at a scan rate of 0.05 Vs^−1^. The measurements were made three times. Then aliquots of a standard UA solution were added into the electrochemical cell and the corresponding voltammograms were recorded. The UA content was calculated considering sample dilution. The absence of matrix effect was measured with the added-found method. Table 4 presents the results of the salivary UA determination on the proposed sensor. It is evident from the data in Table 4 that RSD did not exceed 3.9%. The recovery was 96–105%, which indicates that there was no matrix effect on the analytical signal of UA.

### 3.7. Study of UA and АОА Correlation

AOA of biological fluids is one of the biomarkers of oxidative stress and related diseases [57,58]. It is reported that UA is the main contributor to AOA in plasma/serum (60–80%), saliva (70%) and urine (75%) [57]. Kazakov et al. [59] studied the relationship between UA and AOA in the blood serum of patients with cardiovascular and oncological disorders.

**Figure 7:**
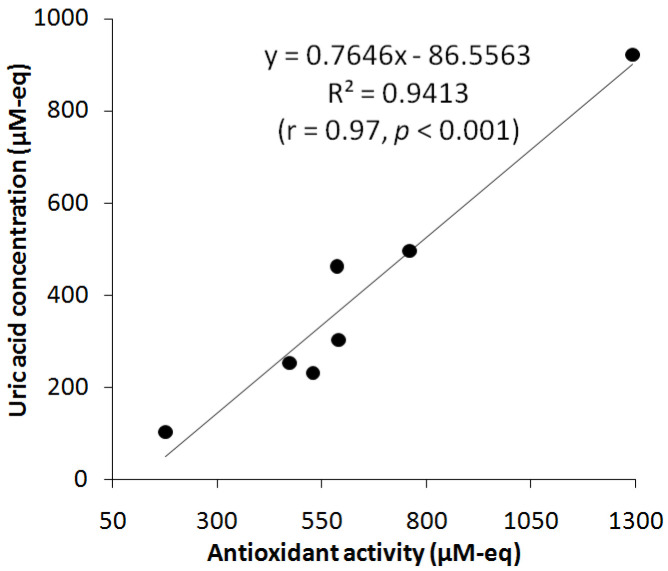
Correlation between salivary UA and AOA.

To determine the contribution of UA in salivary AOA, the correlation between UA and AOA in the saliva of relatively healthy non-smoking participants was studied. Salivary AOA was measured using the potentiometry method outlined in [32]. Summarily, 0.2 mL of the saliva sample were added to 9.8 mL of PB pH 7.2, containing 10 mM K_3_[Fe(CN)_6_] and 0.1 mM K_4_[Fe(CN)_6_]. The changes (decrease) in the potential were registered in the two-electrode cell containing the planar platinum electrode (working electrode) and silver/silver chloride electrode Ag/AgCl/KCl (reference electrode). When correlating salivary UA and AOA, UA concentration was expressed in μM-eq, taking into consideration that two electrons were involved in UA oxidation on CVE_act_ (Scheme 1). As can be seen in Figure 7, there is a correlation between AOA and salivary UA concentration, i.e., higher concentration of UA in saliva leads to higher AOA. The Pearson linear correlation coefficient between salivary UA and AOA is r = 0.9413, *p* < 0.001, which indicates their direct and strong relationship (Figure 7). The contribution of UA to the AOA of the studied saliva samples averaged around 60%, which is consistent with the research literature [1,57].

## 4. Conclusions

The paper describes a new highly efficient and sensitive sensor based on activated carbon veil and used for non-invasive determination of UA. Carbon veil is a material of potential applications. It has a porous structure, effective surface area and good electrical conductivity. A simple, fast, and low-cost hot lamination technology has been exploited for the electrode fabrication. Electrochemical polarization at E = 2.0 V has been used to activate the CVE surface. As a result, the number of surface oxygen-containing functional groups has grown. The sensor has a low detection limit of 0.05 μM and a wide linear range from 0.09 to 700 μM, which enables it to cover the entire range of UA levels in human saliva. The electrode has shown good selectivity with regard to ascorbic acid, creatinine, urea, and glucose. The proposed sensor has been successfully used to determine UA in artificial and human saliva. High recovery (96–105%) has demonstrated that the proposed sensor could be well applied for salivary UA determination. A good correlation (r = 0.97) has been observed between the levels of salivary UA and AOA. It has been found that UA provides about 60% of the total AOA of saliva.

## Figures and Tables

**Table 1:** Elemental composition of CVE and CVE_act_ surfaces with different treatments.

Electrode	Fiber			Binder	
	Weight (C), %	Weight (O), %	Weight (N), %	Weight (C), %	Weight (O), %
CVE	95.96	0.34	3.70	96.02	3.98
CVE_act_ (1.0 V)	96.22	0.30	3.48	96.88	3.12
CVE_act_ (1.6 V)	94.56	3.51	1.93	96.45	3.86
CVE_act_ (2.0 V)	74.23	18.88	6.89	96.05	3.95

**Table 2:** Fitted EIS parameters using a Randles equivalent cell (*n* = 3; *p* = 0.95).

Electrode	R_s_ *, Ohm	R_ct_ **, kOhm	W, μMhos^1/2^
CVE	82.6 ± 1.1	8.21 ± 0.17	387 ± 17
CVE_act_ (1.0 V)	67.8 ± 1.0	6.45 ± 0.14	524 ± 22
CVE_act_ (1.6 V)	58.7 ± 1.4	3.36 ± 0.11	844 ± 31
CVE_act_ (2.0 V)	53.1 ± 0.6	2.96 ± 0.09	690 ± 23

* R_s_—electrolyte resistance; ** R_ct_—charge transfer resistance.

**Table 3:** Comparison of the most recent electrochemical (bio)sensors for the determination of UA in non-invasive biological samples.

Electrode ^1^	Modifier ^2^	Electrolyte ^3^	LR ^4^, μM	LOD ^5^, μM	Method ^6^	Sample	Reference
CVE_act_	–	PB pH 6.0	0.09–700	0.05	LSV	Saliva	This work
CSPE	MWCNTs/UO	ASal. pH 6.8	5–1000	0.33	CA	Saliva	[19]
BDDE	–	BRB pH 2.25	8–1000	7.7	DPV	Urine	[20]
GCE	CuO-NFs	PBS pH 7.4	1–351	0.6	CV	Urine	[21]
GCE	ZIF-11	BRB pH 7.0	50–540	0.48	DP-ASV	Urine	[22]
ITO	PEDOT–GO	ASal. pH 6.8	2–1000	0.75	DPV	Saliva	[45]
CSPE	Ni_0.75_Zn_0.25_(OH)_2_-NPs	ASw. pH 5.0, ASal. pH 6.7	20–170, 200–2000	0.023, 0.023	CA, CV	Sweat, saliva	[46]
CPE	β-CD	AB pH 5.0	10–170	4.5	A	Urine, saliva	[47]
G-PLA-3DPE	–	BRB pH 2.0	0.5–250	0.02	BIA-MPA	Saliva	[48]
Pt	GF-Nf/PANI/GO-UO	PBS pH 7.4	3–300	3	A	Saliva	[49]
PB-CSPE	UO/PPD	ASal. pH 6.7	0–1000	n/a	CA	Saliva	[50]
CSPE	Os-HRP/UO	PBS pH 7.4	10–400	n/a	CA	Saliva	[51]
CSPE	Cr-Au/UO	PBS pH 7.3	0–500	n/a	CA	Saliva	[52]
GSPE	–	AB pH 4.6	3–40	0.74	DPV	Sweat	[53]
GCE	g-C_3_N_4_-NSs	PBS pH 7.4	100–1000	4.45	DPV	Urine	[54]
GCE	ZnPc-rGO-UO-Nf	PB* pH 3.1	0.5–100	0.15	DPV	Urine	[55]
GCE	MC-GO-Fe_3_O_4_-NPs	PB pH 7.0	0.5–140	0.17	DPV	Urine	[56]

^1^ CVE_act_—carbon veil electrode activated at 2.0 V; CSPE—carbon or graphite screen-printed electrode; BDDE—boron-doped diamond electrode; GCE—glassy carbon electrode; ITO—indium tin oxide; CPE—carbon paste electrode; G-PLA-3DPE—3D printed electrode based on graphene (G) and polylactic acid (PLA); PB-CSPE—prussian-blue-graphite screen-printed electrode; GSPE—graphene screen-printed electrode. ^2^ MWCNTs—multi-walled carbon nanotubes; UO—uricase; NFs—nanoflakes; ZIF-11—zeolite imidazole framework-11; PEDOT—poly(3,4-ethylenedioxythiophene); GO—graphene oxide; NPs—nanoparticles; β-CD—b-cyclodextrin; GF—graphene flakes; Nf—nafion; PANI—polyaniline; PPD—poly-o-phenylenediamine; Os-HRP—osmium-wired horseradish peroxidase; g-C_3_N_4_-NSs—graphitic-like carbon nitride nanosheets; ZnPc—zinc tetraaminophthalocyanine; rGO—reduced graphene oxide; MC—methylcellulose. ^3^ PB—phosphate buffer; ASal.—artificial saliva; BRB—Britton-Robinson buffer; PBS—phosphate buffered saline; ASw.—artificial sweat; AB—acetate buffer; PB*—phosphate buffer containing KCl. ^4^ LR—linear range. ^5^ LOD—limit of detection. ^6^ LSV—linear sweep voltammetry; CA—chronoamperometry; DPV—differential-pulse voltammetry; CV—cyclic voltammetry; DP-ASV—differential pulse-anodic stripping voltammetry; A—amperometry; BIA-MPA—multiple-pulse amperometry combined with batch-injection analysis.

**Table 4:** Results of salivary UA determination with the use of the proposed sensor CVE_act_ (*n* = 3, *p* = 0.95).

Sample	Found in Saliva, μM	RSD, %	Added, μM	Found in Saliva with Additive, μM	Found Additive, μM	R, %
Artificial saliva	-	-	200	-	203 ± 7	102
Sample 1	51 ± 5	3.9	100	147 ± 17	96 ± 13	96
Sample 2	115 ± 5	1.7	100	216 ± 9	101 ± 5	101
Sample 3	126 ± 12	3.7	100	223 ± 33	98 ± 9	98
Sample 4	151 ± 9	2.4	100	254 ± 8	103 ± 5	103
Sample 5	230 ± 20	3.4	100	336 ± 20	105 ± 6	105
Sample 6	248 ± 14	2.3	300	553 ± 38	305 ± 10	102
Sample 7	461 ± 11	1.0	500	956 ± 24	495 ± 17	99

## Data Availability

The datasets used and/or analyzed during the current study are available from the corresponding author upon reasonable request.

## Supplementary Materials

The following are available online at https://www.mdpi.com/2079-6374/11/8/287/s1, Figure S1: LS voltammograms of 0.1 mM UA for the CVE activated at 2.0 V (a), Au-gr/CVE (b) and TrX100/CVE (c) in PB pH 6.0. Potential scan rate 0.05 Vs^–1^. (Au-gr—phytosynthesized gold nanoparticles; TrX100—non-ionogenic surfactant Triton X-100); Figure S2: Photo of the CVE; Figure S3: SEM-images and EDS spectrum of fiber and binder of the non-activated CVE (a), CVE activated at 1.6 V (b) and at 2.0 V (c). Inserts: element weight contents (%); Figure S4: Kinetic dependences obtained with the use of CVE_act_ in PB pH 6.0, containing 0.01 mM UA; Figure S5: LS voltammograms of UA with different concentrations (0.09–700 μM) on CVE_act_ in PB pH 6.0 at potential scan rate 0.05 Vs^–1^ (a) and corresponding dependences I_p_ vs. UA concentration (b) (n = 3 for each concentration); Table S1: Interfering influence of some substances on UA determination.

## References

[1] Vernerová A., Kujovská Krčmová L., Melichar B., Švec F. (2021). Non-invasive determination of uric acid in human saliva in the diagnosis of serious disorders. Clin. Chem. Lab. Med..

[2] Choromańska K., Choromańska B., Dąbrowska E., Bączek W., Myśliwiec P., Dadan J., Zalewska A. (2015). Saliva of obese patients – Is it different?. Postepy Hig. Med. Dosw..

[3] Bakhtiari S., Toosi P., Samadi S., Bakhshi M. (2017). Assessment of uric acid level in the saliva of patients with oral lichen planus. Med. Princ. Pract..

[4] Riis J.L., Bryce C.I., Matin M.J., Stebbins J.L., Kornienko O., van Huisstede L., Granger D.A. (2018). The validity, stability, and utility of measuring uric acid in saliva. Biomark. Med..

[5] Bilancio G., Cavallo P., Lombardi C., Guarino E., Cozza V., Giordano F., Palladino G., Cirillo M. (2019). Saliva for assessing creatinine, uric acid, and potassium in nephropathic patients. BMC Nephrol..

[6] Wu W.-C., Chen H.-Y.T., Lin S.-C., Chen H.-Y., Chen F.-R., Chang H.-T., Tseng F.-G. (2019). Nitrogen-doped carbon nanodots prepared from polyethylenimine for fluorometric determination of salivary uric acid. Microchim. Acta.

[7] Liu X.-Y., Luo Y., Zhou C.-Y., Peng A., Liu J.-Y. (2017). A sensitive and accurate method to simultaneously measure uric acid and creatinine in human saliva by using LC-MS/MS. Bioanalysis.

[8] Honeychurch K.C. (2017). The determination of uric acid in human saliva by liquid chromatography with electrochemical detection. J. Anal. Bioanal. Sep. Tech..

[9] Wang X., Lu J., Tang X., Qiu P. (2020). Colorimetric detection of uric acid with high sensitivity using Cu_2_O@Ag nanocomposites. Chem. Afr..

[10] Guan Y., Chu Q., Ye J. (2004). Determination of uric acid in human saliva by capillary electrophoresis with electrochemical detection: Potential application in fast diagnosis of gout. Anal. Bioanal. Chem..

[11] Chu Q.C., Lin M., Geng C.H., Ye J.N. (2007). Determination of uric acid in human saliva and urine using miniaturized capillary electrophoresis with amperometric detection. Chromatographia.

[12] Li Y., Zhang Y.X., Xue W., Zhou Y.J., Duan D.D., Ding Y.P., Zhang R.Z. (2020). A flexible and highly selective nonenzymatic uric acid sensor based on free-standing carbon fiber. Funct. Mater..

[13] Stozhko N., Bukharinova M., Galperin L., Brainina K. (2018). A nanostructured sensor based on gold nanoparticles and nafion for determination of uric acid. Biosensors.

[14] Wang X., Chen S., Tang X., Linc D., Qiu P. (2019). Ultrasensitive detection of uric acid in serum of patients with gout by a new assay based on Pt@Ag nanoflowers. RSC Adv..

[15] Erden P.E., Kılıç E. (2013). A review of enzymatic uric acid biosensors based on amperometric detection. Talanta.

[16] Lakshmi D., Whitcombe M.J., Davis F., Sharma P.S., Prasad B.B. (2011). Electrochemical detection of uric acid in mixed and clinical samples: A review. Electroanalysis.

[17] Ratautaite V., Samukaite-Bubniene U., Plausinaitis D., Boguzaite R., Balciunas D., Ramanaviciene A., Neunert G., Ramanavicius A. (2021). Molecular imprinting technology for determination of uric acid. Int. J. Mol. Sci..

[18] Kaya S.I., Kurbanoglu S., Ozkan S.A. (2019). Nanomaterials-based nanosensors for the simultaneous electrochemical determination of biologically important compounds: Ascorbic acid, uric acid, and dopamine. Crit. Rev. Anal. Chem..

[19] Shi W., Li J., Wu J., Wie Q., Chen C., Bao N., Yu C., Gu H. (2020). An electrochemical biosensor based on multi-wall carbon nanotube–modified screen-printed electrode immobilized by uricase for the detection of salivary uric acid. Anal. Bioanal. Chem..

[20] Cinková K., Kianičková K., Stanković D.M., Vojs M., Martond M., Švorc L. (2018). The doping level of boron-doped diamond electrodes affects the voltammetric sensing of uric acid. Anal. Methods.

[21] Buledi J.A., Ameen S., Memon S.A., Fatima A., Solangi A.R., Mallah A., Karimi F., Malakmohammadi S., Agarwal S., Gupta V.K. (2021). An improved non-enzymatic electrochemical sensor amplified with CuO nanostructures for sensitive determination of uric acid. Open Chem..

[22] Thanh T.S., Qui P.T., Tu N.T.T., Toan T.T.T., Hoa T.T.B., Son L.V.T., Nguyen D.M., Tuyen T.N., Khieu D.Q. (2021). Electrochemical determination of uric acid in urine by using zeolite imidazolate framework-11 modified electrode. J. Nanomater..

[23] Bhatt P., Goe A. (2017). Carbon fibres: Production, properties and potential use. Mat. Sci. Res. India.

[24] Kumar N., Gangwar A.K., Devi K.S., Khanna R., Cayumil R. (2018). Carbon fibers in biomedical applications. Recent Developments in the Field of Carbon Fibers.

[25] Torrinha A., Morais S. (2021). Electrochemical (bio)sensors based on carbon cloth and carbon paper: An overview. TrAC Trends Anal. Chem..

[26] Stozhko N.Y., Bukharinova M.A., Khamzina E.I., Tarasov A.V., Sokolkov S.V. (2020). Film carbon veil-based electrode modified with Triton X-100 for nitrite determination. Chemosensors.

[27] Zhu W., Zhang Y., Gong J., Ma Y., Sun J., Li T., Wang J. (2019). Surface engineering of carbon fiber paper toward exceptionally high-performance and stable electrochemical nitrite sensing. ACS Sens..

[28] Brainina K.Z., Bukharinova M.A., Stozhko N.Y., Sokolkov S.V., Tarasov A.V., Vidrevich M.B. (2020). Electrochemical sensor based on a carbon veil modified by phytosynthesized gold nanoparticles for determination of ascorbic acid. Sensors.

[29] Torrinha Á., Martins M., Tavares M., Delerue-Matos C., Morais S. (2021). Carbon paper as a promising sensing material: Characterization and electroanalysis of ketoprofen in wastewater and fish. Talanta.

[30] Tarasov A.V., Khamzina E.I., Bukharinova M.A., Stozhko N.Y. (2021). Flexible potentiometric sensor system for non-invasive determination of antioxidant activity of human skin: Application for evaluating the effectiveness of phytocosmetic products. Chemosensors.

[31] Benjamin O., Silcock P., Beauchamp J., Buettner A., Everett D.W. (2013). Volatile release and structural stability of b-lactoglobulin primary and multilayer emulsions under simulated oral conditions. Food Chem..

[32] Brainina K.Z., Varzakova D.P., Kazakov Y.E., Vidrevich M.B. (2018). Noninvasive electrochemical antioxidant activity estimation: Saliva analysis. Biointerface Res. Appl. Chem..

[33] Burns D.T., Danzer K., Townshend A. (2002). Use of the terms “recovery” and “apparent recovery” in analytical procedures (IUPAC Recommendations 2002). Pure Appl. Chem..

[34] Zhu W., Zhang X., Yin Y., Qin Y., Zhang J., Wang Q. (2018). In-situ electrochemical activation of carbon fiber paper for the highly efficient electroreduction of concentrated nitric acid. Electrochim. Acta.

[35] Kakhki R.M. (2019). A review to recent developments in modification of carbon fiber electrodes. Arab. J. Chem..

[36] Tukimin N., Abdullah J., Sulaiman Y. (2018). Electrodeposition of poly(3,4-ethylenedioxythiophene)/reduced graphene oxide/manganese dioxide for simultaneous detection of uric acid, dopamine and ascorbic acid. J. Electroanal. Chem..

[37] Wang Z., Han Y., Zeng Y., Qie Y., Wang Y., Zheng D., Lu X., Tong Y. (2016). Activated carbon fiber paper with exceptional capacitive performance as a robust electrode for supercapacitors. J. Mater. Chem. A.

[38] Wei Y., Xu Z., Wang S., Liu Y., Zhang D., Fang Y. (2020). One-step preparation of carbon quantum dots-reduced graphene oxide nanocomposite–modified glass carbon electrode for the simultaneous detection of ascorbic acid, dopamine, and uric acid. Ionics.

[39] Motshakeri M., Travas-Sejdic J., Phillips A.R.J., Kilmartin P.A. (2018). Rapid electroanalysis of uric acid and ascorbic acid using a poly(3,4-ethylenedioxythiophene)-modified sensor with application to milk. Electrochim. Acta.

[40] Shekh M.I., Amirian J., Du B., Kumar A., Sharma G., Stadler F.J., Song J. (2020). Electrospun ferric ceria nanofibers blended with MWCNTs for high-performance electrochemical detection of uric acid. Ceram. Int..

[41] Golovanov S.A., Sivkov A.V., Polikarpova A.M., Drozhzheva V.V., Andryuhin M.I., Prosyannikov M.Y. (2018). Metabolic risk factors and formation of urinary stones. Study III: Effect of urine pH. Exp. Clin. Urol..

[42] Bard A.J., Faulkner L.R. (2001). Electrochemical Methods: Fundamentals and Applications.

[43] Shibasaki K., Kimura M., Ikarashi R., Yamaguchi A., Watanabe T. (2012). Uric acid concentration in saliva and its changes with the patients receiving treatment for hyperuricemia. Metabolomics.

[44] Mäkilä E., Kirveskari P. (1969). A study of ascorbic acid in human saliva. Arch. Oral. Biol..

[45] Huang X., Shi W., Li J., Bao N., Yu C., Gu H. (2020). Determination of salivary uric acid by using poly(3,4-ethylenedioxythipohene) and graphene oxide in a disposable paper-based analytical device. Anal. Chim. Acta.

[46] Azeredo N.F.B., Gonçalves J.M., Rossini P.O., Araki K., Wang J., Angnes L. (2020). Uric acid electrochemical sensing in biofluids based on Ni/Zn hydroxide nanocatalyst. Microchim. Acta.

[47] Ramírez-Berriozabal M., Galicia L., Gutiérrez-Granados S., Cortes J.S., Herrasti P. (2008). Selective electrochemical determination of uric acid in the presence of ascorbic acid using a carbon paste electrode modified with β-Cyclodextrin. Electroanalysis.

[48] Cardoso R.M., Silva P.R.L., Lima A.P., Rocha D.P., Oliveira T.C., do Prado T.M., Fava E.L., Fatibello-Filho O., Richter E.M., Muñoz R.A.A. (2020). 3D-Printed graphene/polylactic acid electrode for bioanalysis: Biosensing of glucose and simultaneous determination of uric acid and nitrite in biological fluids. Sens. Actuators B.

[49] Liao C., Mak C., Zhang M., Chan H.L., Yan F. (2015). Flexible organic electrochemical transistors for highly selective enzyme biosensors and used for saliva testing. Adv. Mater..

[50] Kim J., Imani S., de Araujo W.R., Warchall J., Valdés-Ramírez G., Paixão T.R.L.C., Mercier P.P., Wang J. (2015). Wearable salivary uric acid mouthguard biosensor with integrated wireless electronics. Biosens. Bioelectron..

[51] Kudo H., Takagi Y. (2018). Electrochemical biosensor for simplified determination of salivary uric acid. Sens. Mater..

[52] Liu Z., Chen Y., Zhang M., Sun T., Li K., Han S., Chen H.-J. (2021). Novel portable sensing system with integrated multifunctionality for accurate detection of salivary uric acid. Biosensors.

[53] Yang Y., Song Y., Bo X., Min J., Pak O.S., Zhu L., Wang M., Tu J., Kogan A., Zhang H. (2020). A laser-engraved wearable sensor for sensitive detection of uric acid and tyrosine in sweat. Nat. Biotechnol..

[54] Murugan N., Chan-Park M.B., Sundramoorthy A.K. (2019). Electrochemical detection of uric acid on exfoliated nanosheets of graphitic-like carbon nitride (g-C_3_N_4_) based sensor. J. Electrochem. Soc..

[55] Shi Y.-M., Mei L., Zhang J.-X., Hu K., Zhang X., Li Z.-Z., Miao M.-S., Li X.-M. (2020). Synthesis of zinc tetraaminophthalocyanine functionalized graphene nanosheets as an enhanced material for sensitive electrochemical determination of uric acid. Electroanalysis.

[56] Sohouli E., Khosrowshahi E.M., Radi P., Naghian E., Rahimi-Nasrabadi M., Ahmadi F. (2020). Electrochemical sensor based on modified methylcellulose by graphene oxide and Fe_3_O_4_ nanoparticles: Application in the analysis of uric acid content in urine. J. Electroanal. Chem..

[57] Marrocco I., Altieri F., Peluso I. (2017). Measurement and clinical significance of biomarkers of oxidative stress in humans. Oxid. Med. Cell. Longev..

[58] Tóthová L., Kamodyová N., Červenka T., Celec P. (2015). Salivary markers of oxidative stress in oral diseases. Front. Cell. Infect. Microbiol..

[59] Kazakov Y., Tarasov A., Alyoshina L., Brainina K. (2020). Interplay between antioxidant activity, health and disease. Biointerface Res. Appl. Chem..

