# Supplementary material for: Developing Activated Carbon Veil Electrode for Sensing Salivary Uric Acid
Source: Biosensors (Basel). 2021 Aug 20;11(8):287. doi: 10.3390/bios11080287 (PMC8394272; doi:10.3390/bios11080287)
Supplement: Supplementary file 1 [file biosensors-11-00287-s001.zip › biosensors-1316840-supplementary.pdf]

Supplementary Material

Developing Activated Carbon Veil Electrode for Sensing Salivary Uric Acid

Maria A. Bukharinova, Natalia Yu. Stozhko *, Elizaveta A. Novakovskaya, Ekaterina I. Khamzina, Aleksey V. Tarasov and Sergey V. Sokolkov

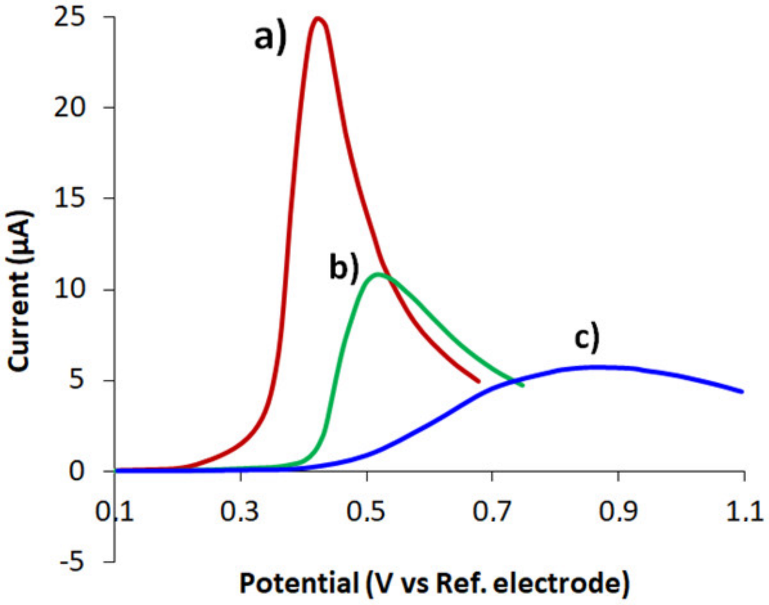

Figure S1. LS voltammograms of 0.1 mM UA for the CVE activated at 2.0 V (**a**), Au-gr/CVE (**b**) and TrX100/CVE (**c**) in PB pH 6.0. Potential scan rate 0.05 Vs^{-1} (Au-gr − phytosynthesized gold nanoparticles; TrX100 − non-ionogenic surfactant Triton X-100).

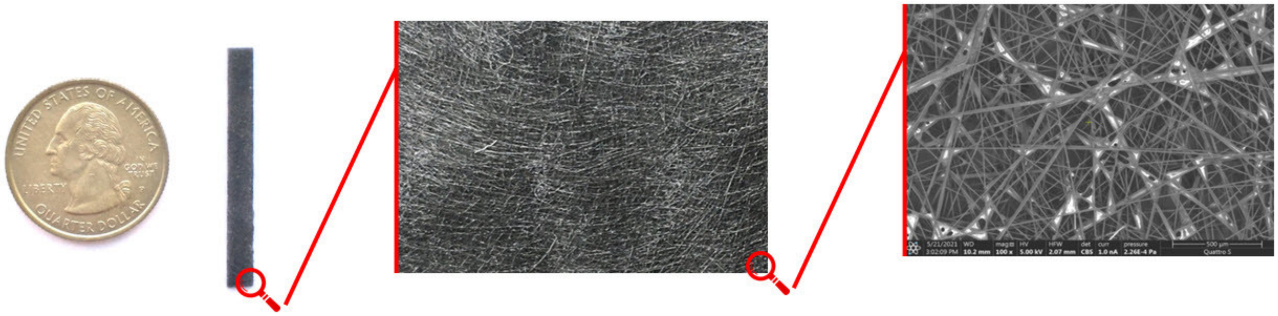

Figure S2. Photo of the CVE.

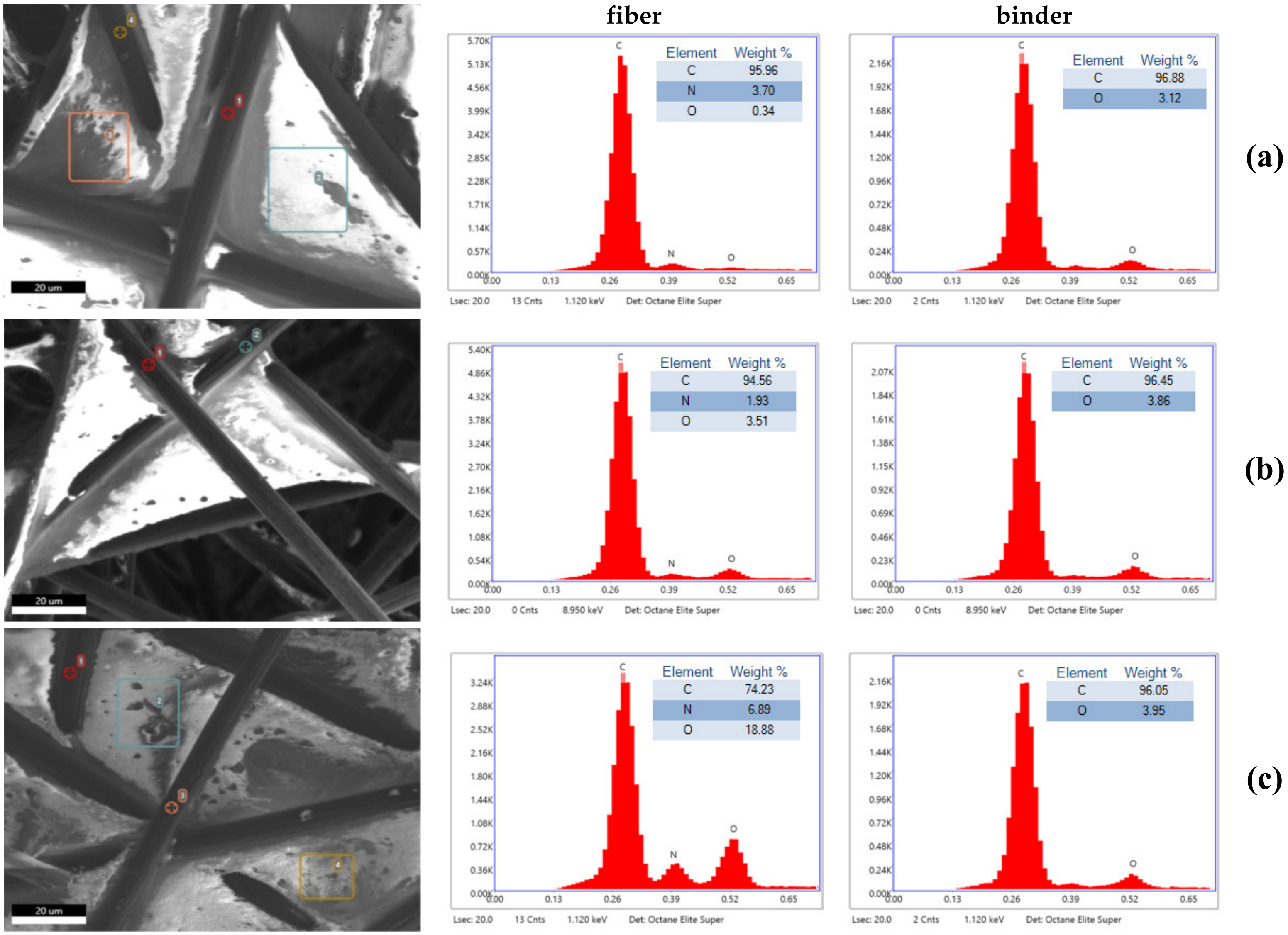

Figure S3. SEM-images and EDS spectrum of fiber and binder of the non-activated CVE (a), CVE activated at 1.6 V (b) and at 2.0 V (c). Inserts: element weight contents (%).

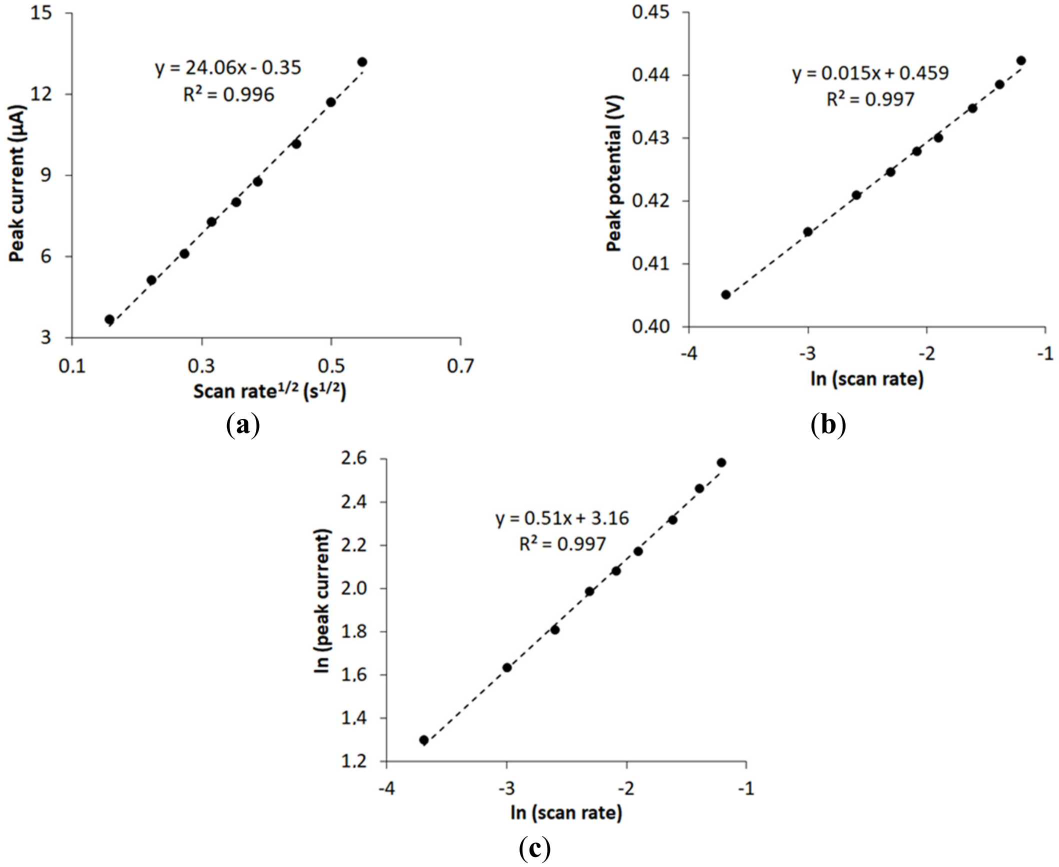

Figure S4. Kinetic dependences obtained with the use of CVE_{act} in PB pH 6.0, containing 0.01 mM UA.

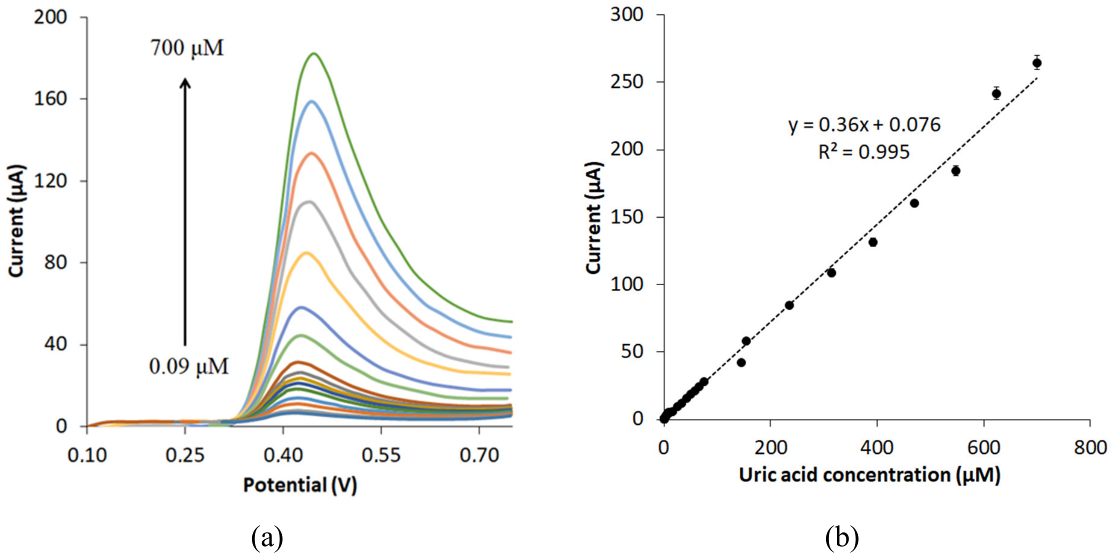

Figure S5. LS voltammograms of UA with different concentrations (0.09–700 μM) on CVE_{act} in PB pH 6.0 at potential scan rate 0.05 Vs^{-1} (a) and corresponding dependences I_p vs. UA concentration (b) (n = 3 for each concentration).

Table S1. Interfering influence of some substances on UA determination.

Interfering Substance	Concentration of Interfering Substance, μM	Added UA, μM	Found UA, μM	R, %
Creatinine	1000	1	1.00±0.09	100
Urea	1000	1	1.05±0.10	105
Glucose	1000	1	1.07±0.08	107
Ascorbic acid	10	1	0.96±0.11	96
